# Key targets of signal transduction neural mechanisms in acupuncture treatment of cardiovascular diseases: Hypothalamus and autonomic nervous system

**DOI:** 10.1016/j.heliyon.2024.e38197

**Published:** 2024-09-20

**Authors:** Xiang Zhou, Jie Zhou, Fan Zhang, Qi Shu, Yan Wu, Hui-min Chang, Bin Zhang, Rong-lin Cai, Qing Yu

**Affiliations:** a College of Acupuncture and Moxibustion, Anhui University of Chinese Medicine, Hefei, 230038, Anhui Province, China; b Anhui Wannan Rehabilitation Hospital (The Fifth People's Hospital of Wuhu), Wuhu, 241000, Anhui Province, China; c Institute of Acupuncture and Meridian Research, Anhui Academy of Chinese Medicine, Hefei, 230038, Anhui Province, China; d Anhui Province Key Laboratory of Meridian Viscera Correlationship, Hefei, 230038, China; e Center for Xin'an Medicine and Modernization of Traditional Chinese Medicine of IHM, Hefei, 230038, China

**Keywords:** Acupuncture, Cardiovascular disease, Acupuncture signal, Autonomic nervous system, Hypothalamus

## Abstract

**Background:**

Cardiovascular disease is the leading cause of death worldwide. As a traditional Chinese treatment method, acupuncture has a unique role in restoring the balance of the human body environment. Due to its safety, non-invasive nature, and effectiveness in treating cardiovascular diseases, acupuncture has been widely welcomed and recognized among the world. A large amount of evidence shows that acupuncture can effectively regulate cardiovascular diseases through the autonomic nervous system. The hypothalamus, as an important component of regulating the autonomic nervous system, plays an important role in regulating the internal environment, maintaining homeostasis, and preserving physiological balance. However, there is currently a scarcity of review articles on acupuncture signal transduction and acupuncture improving cardiovascular disease through the hypothalamus and autonomic nervous system.

**Objective:**

This review delves into the transduction of acupuncture signals and their neural regulatory mechanisms on the hypothalamus and autonomic nervous system, elucidating their impact on cardiovascular disease.

**Methods:**

Review the basic and clinical studies on acupuncture signal transduction mechanisms and the role of the hypothalamus and ANS in acupuncture treatment of cardiovascular diseases published in four English databases (PubMed, Web of Science, MEDLINE, and Springer Cochrane Library) and two Chinese databases (Wanfang Database and China National Knowledge Infrastructure Database) over the past 20 years.

**Results:**

Through sensory stimulation, acupuncture effectively transmits signals from the periphery to the hypothalamus, where they are integrated, and finally regulate the autonomic nervous system to treat cardiovascular diseases.

**Discussion:**

Acupuncture exhibits significant potential as a therapeutic modality for cardiovascular diseases by orchestrating autonomic nervous system regulation via the hypothalamus, thereby gifting novel perspectives and methodologies for the prevention and treatment of cardiovascular ailments.

## Highlights

• Acupuncture signals are transmitted from the periphery stimuli to the hypothalamus.
• Acupuncture regulates the ANS for the treatment of cardiovascular diseases.
• Acupuncture improves cardiovascular disease by hypothalamic regulation of ANS.

## Graphical abstract

**Figure:**
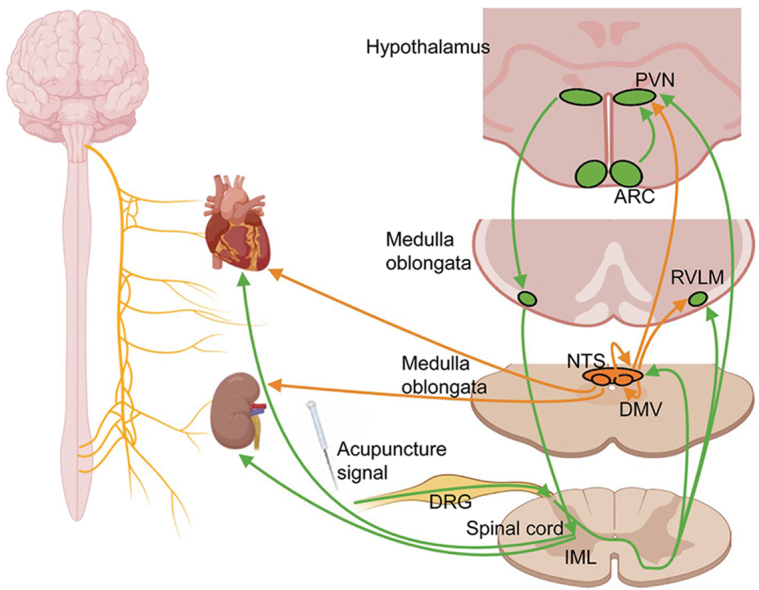

## 1 Introduction

Cardiovascular disease is one of the major causes of death worldwide, posing a serious threat to human health [1]. According to the World Health Organization, approximately 17.9 million people die from cardiovascular disease each year, accounting for 31 % of global deaths [2]. At present, drug therapy [3], exercise therapy intervention [4], and body temperature pretreatment [5] are widely recognized internationally. In addition, timely and effective vascular reconstruction techniques [5] such as percutaneous coronary intervention [6] and coronary artery bypass grafting [7] have been widely used in clinical practice to rescue ischemic myocardium [8], saving patients' lives to a certain extent. However, the side effects associated with drug and surgical treatments, as well as the ischemia-reperfusion process, may further damage the ultrastructure, metabolism, and functionality of myocardial cells, leading to further cell death and up to 50 % myocardial injury in severe cases [9,10].

Acupuncture has been used as a complementary and alternative therapy in the treatment of cardiovascular disease in China for nearly 2000 years, and spread to the world [11,12]. Since 1975, there have been over 10000 randomized controlled trials on acupuncture [13,14]. Numerous clinical studies have shown that acupuncture can be used as an adjunctive intervention to effectively treat cardiovascular disease [15,16]. According to traditional Chinese medicine theory, the effect of acupuncture is attributed to stimulating specific body regions (acupoints) along the meridians to regulate body functions [17]. Especially in the treatment of cardiovascular diseases, the mechanism of acupuncture is generally considered to maintain the stability of the internal and external environment and promote the body to achieve balance [18]. Its main function is to activate peripheral afferent nerves, transmit sensory information from the spinal cord to the brain, activate peripheral autonomic nervous pathways, and ultimately regulate cardiovascular function [[19], [20], [21]]. These cardiac and peripheral vascular pathologies mainly cover conditions such as Myocardial Ischemia [22], Myocardial Ischemia reperfusion injury (MIRI) [23], arrhythmias [24,25], angina pectoris [26,27], heart failure [28] and hypertensive heart disease [29].

In the past several decades, a growing body of research has provided compelling evidence for the significant role of the nervous system in improving cardiovascular diseases [30,31]. The intricate orchestration of cardiac contractions is a result of the concerted interplay between neural and humoral factors, with the neural system assuming a predominant role [32]. The neural regulation of cardiovascular diseases depends on the autonomic nervous system (ANS) and central nervous system (CNS) [33], the hypothalamus in the CNS is the subcortical autonomic nervous center, mainly involved in the regulation of ANS [34]. In recent years, the regulation of the ANS by the hypothalamus is considered an important target for acupuncture treatment of cardiovascular diseases [35]. An increasing number of studies have shown that acupuncture can treat cardiovascular diseases through the hypothalamus [36]. At the same time, how acupuncture signals are transmitted to the hypothalamus has become one of the hot spots of modern scholars [37].

Currently, most reviews focus on how acupuncture regulates the ANS to treat cardiovascular diseases [38]. However, the mechanism of acupuncture participating in the treatment of cardiovascular diseases through hypothalamus has not been systematically summarized, and there is also a lack of review on the neural pathway of acupuncture signal conduction. Therefore, this review comprehensively elaborated the process of acupuncture sensory signal transduction to the hypothalamus, and discussed the research progress of the ANS and hypothalamus in the treatment of cardiovascular diseases by acupuncture. The aim of this paper is to explore the signal transduction pathway and related neurophysiological mechanism of acupuncture in regulating cardiovascular disease, with particular emphasis on the key role of the ANS and hypothalamus.

## 2 Methods

We reviewed the basic and clinical studies on acupuncture signal transduction mechanisms and the role of the hypothalamus and ANS in acupuncture treatment of cardiovascular diseases published in four English databases (PubMed, Web of Science, MEDLINE, and Springer Cochrane Library) and two Chinese databases (Wanfang Database and China National Knowledge Infrastructure Database) over the past 20 years, and organized data on changes in blood pressure（BP） and heart rate （HR）caused by acupuncture under physiological and pathological conditions.

## 3 The transduction of acupuncture signals in cardiovascular diseases

Acupuncture involves the insertion and stimulation of sensory nerve fibers located beneath the skin, within the muscles, or on the periosteum, resulting in somatosensory input signals [[39], [40], [41]]. These input nerve fibers primarily consist of groups I, II, III, and IV (corresponding to Aɑ, β, δ, and C fibers, respectively). It has been reported that manual acupuncture (MA) can stimulate multiple groups of input fibers [42]. During electroacupuncture (EA), the afferent nerve pathways are mainly composed of somatosensory group III and IV fibers, which require a certain level of stimulation intensity [43]. The reflex pathway that reduces HR by needle stimulation consists mainly of group IV muscle afferent fibres, whose activity (even at very low activity rates) activates brainstem γ-aminobutyric acid (GABA) neurons and inhibits sympathetic nervous system (SNS) outflow to the heart [44].

### 3.1 The molecular mechanism of acupuncture signals in cardiovascular diseases

Highly proton-sensitive Acid-sensing ion channels (ASICs) mainly exist in peripheral sensory neurons and are widely expressed in skin and muscle tissues [45], participating in various senses including pain, mechanical stimulation, and chemical perception [46]. TRPV1 serves as the responsive channel for acupuncture [47], and it is the principal channel within sensory nerve fibers for detecting and integrating noxious chemical and thermal stimuli [48]. Recent studies have shown that ASIC3 is widely distributed throughout the entire hypothalamus [49]. Therefore, acupuncture may regulate the hypothalamus through ASIC3 to alleviate cardiovascular disease.

Moreover, acupoints exhibit rich distribution in peripheral sensory nerve endings, and stimulation of these acupoints can regulate the signal transduction of transient receptor potential vanillin like protein 1 (TRPV1) in local tissues and spinal dorsal horn [50]. These afferent signals ascend from the spinal dorsal horn cells to the hypothalamus, thereby promoting the release of neuropeptides and hormones [51]. Research has shown that EA can downregulate TRPV1, thereby exerting analgesic effects [52]. Similar studies have found that EA can exert analgesic effects on rats by regulating the expression of TRPV1 in primary sensory neurons [53]. Apart from its expression in sensory nerve endings, TRPV1 is also widely expressed in the cardiovascular system, and these nerve fibers innervate sensory neurons in cardiac tissue such as the myocardium and epicardial surface [54]. The latest study also revealed the expression of TRPV1 in the paraventricular nucleus (PVN) of the hypothalamus in adult mice [55], and its involvement in the C-fibers vagus sensory pathway in the PVN [56]. Based on the above research, acupuncture signals transmit sensory stimuli to the hypothalamus for integration through ASIC3 and TRPV1, and then regulate the ANS to protect cardiovascular diseases.

### 3.2 The neural pathway of acupuncture signals in cardiovascular diseases

In recent years, the CNS mechanism of acupuncture has become a hot research topic, mainly focusing on the neural pathway transduction mechanism of acupuncture signals [17]. Experimental studies have shown that after injecting cholera toxin B subunit into the tissues located at acupoints in the rat forehead and face, its related markers were distributed in the cervical dorsal root ganglia (DRG) [57]. Most DRG neurons innervating ST36 acupoints (overlies the deep peroneal nerve and tibialis anterior muscle, to a depth of 5 mm) are interneurons with a single spike discharge mode, which mediates acupuncture signals [58]. Visceral and median nerve stimulation activates premotor sympathetic neurons located in the intermediolateral nucleus (IML) of the thoracic spinal cord. Low frequency EA and MA stimulation can effectively inhibit the premotor sympathetic cardiovascular neurons in the rostral ventrolateral medulla (RVLM) where visceral and median nerves converge and input [59]. RVLM neurons, on the other hand, mainly receive inputs from the PVN of the hypothalamus and the nucleus tractus solitarius (NTS) of the brainstem, and activate IML neurons [60]. Moreover, acupuncture exerts an antihypertensive effect via increasing the expression of nitric oxide synthase in neurons of the arcuate nucleus (ARC) of spontaneously hypertensive rats [61]. This indicates that ARC may be an key target for acupuncture to regulate BP. Further research has found that the regulatory effect of EA on BP response induced by visceral organ stimulation is related to the inhibition of cardiovascular neurons from the ARC to the RVLM through opioid mechanisms [62].

Clinical studies have shown that MA in the auricular branch of the vagus nerve can activate the nodose ganglion (NG) in the caudal part of the vagus nerve [63]. Triggering of central vagal effects is associated with the dorsal vagal complex (DVC), which consists of three components: the NTS, the dorsal motor nucleus of the vagus complex (DMV) and the area postrema [35]. DMV contains various neurotransmitters and corresponding receptors with extensive fibre connections to central and peripheral regions. Its neurons can directly sense information in peripheral blood and cerebrospinal fluid [64]. The glutamate released from the vagus nerve fibers of DMV can activate the secondary neurons of NTS, which then GABA receptor serves as a mediator to transmit information to the DMV, causing an increase in DMV activity. Finally, through the DMV efflux pathway, it releases the corresponding neurotransmitters, such as acetylcholine, thereby regulating the heart [65]. Studies have shown that acupuncture signals can accumulate in DVC [66], effectively regulating the number of c-Fos^+^ cells in the DMV [67], activating DMV neurons. Interestingly, injection of GABA receptor blockers into the NTS region reversed the modulatory effect of EA on bradycardia [68]. This suggests that EA inhibits vagally evoked NTS activity via a GABAergic mechanism that may involve projections from glutamatergic nucleus to NTS neurons.

**Fig. 1:**
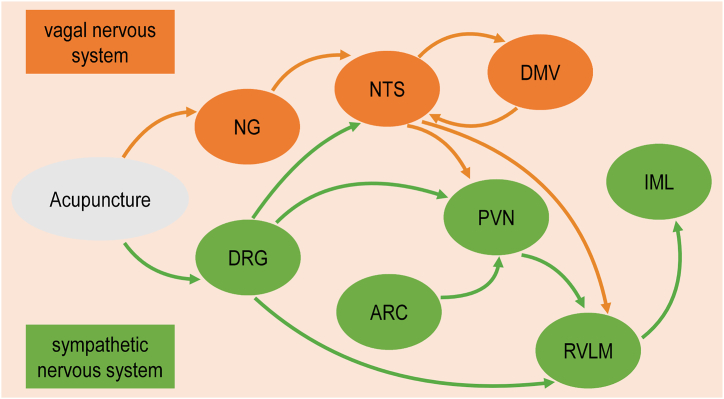
Schematic diagram of neural pathways for acupuncture signal transduction.

In summary, acupuncture stimulates peripheral sensory endings, transmitting stimulation signals from DRG or NG to the medulla oblongata and then to the hypothalamus for integration, thereby regulating ANS to protect cardiovascular function (Fig. 1).

## 4 Current situation and analysis of acupuncture regulating the autonomic nervous system in the treatment of cardiovascular diseases

ANS [69], also referred to as the vegetative or visceral nervous system, plays a crucial role in regulating the functional activities of visceral organs and maintaining relative balance and stability within the body's internal and external environments [70]. As such, the ANS has been a focal point in acupuncture research [71]. Myocardial ischemia induces alterations in the ANS, affecting the dynamic balance between the SNS and the vagal nervous system (VNS) [72]. Specifically, when the neural network of the heart is remodeled following myocardial ischemia, EA can increase left ventricular ejection fraction and fractional shortening, thereby reducing infarct size [73]. This provides direct evidence for the beneficial regulation of acupuncture on cardiac neural tissue remodeling.

Clinical studies have shown that PC6 (between the palmaris longus tendon and the flexor carpi radialis tendon) and ST36 both affected cardiac activities in healthy volunteers [74]. Hypertension is considered another modifiable risk factor for cardiovascular disease, stroke, renal failure, and death, and it can also cause dysfunction of the ANS [75]. Clinical studies have also shown that low current, low-frequency EA can reduce BP in patients with mild to moderate hypertension [76].

Additionally, experimental studies have shown that low-frequency EA has superior effects on three ANS indicators compared to medium-frequency EA: heart rate variability (HRV) improved by 61 %, SNS tension decreased by 42 %, and parasympathetic nerve tension increased by 56 %. Low-frequency EA attenuated the stress response of the SNS, improved vagal nerve tension and HRV [77]. These results suggest that EA has a regulatory effect on the ANS, especially in terms of HRV, VNS and SNS.

### 4.1 Effect of acupuncture on heart rate variability

HRV analysis is increasingly being utilized to evaluate autonomic function [78]. It regulated by the neurohumoral system, is an important indicator for evaluating and preventing cardiovascular diseases [79,80]. In clinical practice, HRV analysis provides insights into the activity, balance, and pathological conditions of the cardiac ANS [81]. Acupuncture has shown outstanding effects on the ANS function of the heart, increasing the tension of the parasympathetic nervous system and improving HRV levels [82,83]. The reduced HR response induced by acupuncture is thought to be the result of the coordinated interaction between increased cardiac vagal activity and decreased cardiac sympathetic activity [84]. Acupoint stimulation can significantly reduce HR, HRV, and the ratio of low-frequency to high-frequency [85].

Nishijo et al. concluded that acupuncture reduces heart rate by promoting cardiac vagus nerve activity and inhibiting cardiac sympathetic nerve activity [86]. Minagawa, M. et al. observed that stimulation at RN12 (midpoint of the line connecting the umbilicus and xiphoid process) increased the power of LF and HF components, an effect indicating cardiac vagal activation [87]. Clinical studies have shown that compared to acupuncture at CV16 (midpoint of the line connecting the two nipples), acupuncture at CV17 (the anterior midline, level the 5th intercostal space) is more effective in reducing HR and increasing the high-frequency component power of HRV [88]. Specifically, VNS activity increased by 17 % following acupuncture [89], highlighting the role of acupuncture in stabilizing cardiac autonomic function in patients with ischemic heart disease and its impact on HRV. Another clinical study suggests that acupuncture at specific acupoints on the hand can effectively lower HR, enhance activity of the VNS, and decrease the VNS-to-SNS ratio, thereby regulating cardiac autonomic function [90]. As a result, it is clinically found that acupuncture can reduce mean arterial pressure and HR by inhibiting SNS [91].

There are studies reporting that EA regulates gastric distension induced reflex sympathetic inhibition and vagus nerve excitation through the GABAA receptor mechanism in RVLM, thereby reducing HR and BP [92]. This improvement mechanism may be associated with the attenuation of excessive SNS excitation and the regulation of SNS and VNS balance. Moreover, similar studies have shown that stimulation of acupoint HT7 (the radial depression of the flexor carpi ulnaris tendon) can activate the parasympathetic nervous system, augmenting the anti-arrhythmic effects in the regions governed by HRV, consequently reducing the incidence of atrial fibrillation [93].

Thus, acupuncture could potentially improve cardiovascular diseases by modulating the relative activity of the SNS and the VNS, leading to decreased HRV and improved cardiovascular function.

### 4.2 Effect of acupuncture on the vagus nerve

The release of acetylcholine from postganglionic fiber endings of the cardiac VNS can lead to a decrease in HR by acting on M-type cholinergic receptors on the myocardial cell membrane [94]. EA exerts its cardioprotective effects through the vagus nerve mediated cholinergic pathway [95].Clinical studies have shown that EA at PC6 can induce co activation of cardiac vagus nerve and muscle SNS to alleviate left ventricular dysfunction and reduce inflammation [96]. Additionally, EA can directly inhibit the activity of ventricular muscles and promote vasodilation [97]. Takahashi K et al. indicated that EA stimulation with 2 Hz near near the cervical sympathetic trunk increased parasympathetic activity and decreased HR [98]. Hamvas et al. pointed out that in terms of increasing vagus nerve tension, real acupuncture has better effect than placebo acupuncture, and can improve health [99]. A study found that acupuncture at HT7 acupoint can induce greater excitability in VNS compared to CV16 acupoint [100]. These findings collectively suggest that acupuncture targeting multiple specific acupoints can effectively regulate VNS and improve cardiovascular disease.

In addition, auricular acupuncture, as a safe and effective non drug therapy, is a representative of acupuncture [101]. Modern anatomy shows that concha is the sole area with vagus nerve distribution on the body surface [102,103], which overlaps the visceral distribution area of auricular points in traditional Chinese medicine. Wang et al. has proposed transcutaneous auricular vagus nerve stimulation by combining auricular nerve anatomy, vagus nerve stimulation and traditional auricular acupuncture therapy [104].

Acupuncture in the auricular vagus nerve region has also been reported to reduce narcotic and alcohol withdrawal symptoms, with the underlying physiological mechanism described as increased parasympathetic activity [105]. Research has shown that transcutaneous auricular vagus nerve stimulation has a protective effect on the heart, with neovascularization playing a crucial role in the restoration of myocardial blood supply [106]. Particularly during the early stages following MIRI, transcutaneous auricular vagus nerve stimulation has been found to upregulate the expression of vascular endothelial growth factor, endothelial nitric oxide, and fibroblast growth factor in the ischemic area, thereby enhancing angiogenesis [107]. Stimulation of the auricular branch of the vagus nerve may elicit cardiovascular responses, suggesting that transcutaneous auricular vagus nerve stimulation may be involved in the regulation of cardiovascular reactions [108]. Therefore, transcutaneous auricular vagus nerve stimulation can not only enhance VNS, but also regulate cardiovascular function and improve cardiovascular disease.

### 4.3 Effect of acupuncture on sympathetic nerves

The SNS is primarily responsible for regulating cardiovascular function by releasing NE and binding to myocardial receptors [109,110]. The selective activation of cardiac chemical receptors can affect the activity of most cells in PVN and regulate SNS tension. This indicates an interaction between the hypothalamus and the ANS [111]. Regional differences in sympathetic innervation correspond to different areas of influence over cardiac function that cooperate to effectively control cardiac performance [112]. Acupuncture can be used to treat cardiovascular diseases related to SNS activity, such as myocardial ischemia and hypertension [113]. It can reverse the state of cardiac autonomic remodeling following myocardial infarction. It achieves this by inhibiting sympathetic reinnervation in the peri-infarct region, thereby enhancing cardiac function [114].

GV20 (at the midpoint of the line connection the apexes of both ears) has been found to have the ability to vasodilate and reduce sympathetic activity in response to stress [[115], [116], [117], [118], [119]]. As a result, acupuncture at GV20 acupoint can reduce HR. A recent study applied left common peroneal nerve microneurography to investigate the effects of acupuncture on postganglionic SNS activity in humans, revealing its significant impact on both HR and BP [120]. E Haker et al. found that the superficial needle insertion into the skin overlaying the right thenar muscle caused a pronounced balanced increase in both the sympathetic and parasympathetic activity [121]. Acupuncture in healthy individuals is related to changes in the activity of the sympathetic and parasympathetic nervous systems, depending on the stimulation site and observation time. Uchida et al. pointed out the neural mechanism by which pinprick stimulation induces inhibition of the HR reflex. Interestingly, the magnitude of sympatho-inhibitory response to acupuncture-like stimulation does not depend on pre-existing sympathetic tone [122]. Similarly, Michikami et al. found that EA stimulation resets the arterial baroreflex neural arc to lower sympathetic nerve activity [123]. It suggests that acupuncture may regulate HR and BP by modulating the activity of the SNS.

Acupuncture pretreatment inhibited the expression of related growth protein-43, reduced excessive sprouting of SNS, and increased the distribution density of positive fibers, exhibiting anti-inflammatory effects and affecting SNS remodeling. By alleviating the secondary physiological changes caused by heterogeneous remodeling, this method has the potential to prevent and treat MIRI [124]. Similar studies have shown that EA may enhance the expression of neuromodulatory protein-1 in myocardial tissue, while reducing the expression of tyrosine hydroxylase and growth-associated protein-43, thereby inhibiting sympathetic hyperexcitation after myocardial infarction and achieving myocardial protection [125]. These findings suggest that acupuncture can protect cardiac function by regulating the expression of SNS related proteins in myocardial tissue. Experimental studies have shown that EA at PC6 acupoint can reduce sympathetic nervous activity in the heart and improve cardiac function in rats with myocardial ischemia [126].

A recent study using direct nerves recording the sympathetic cervical ganglia of the heart showed that EA pretreatment can inhibit the frequency of sympathetic nerve discharges [127]. Another study indicating that acupuncture at the same spinal segment of the acupoint inhibits the somatorenal sympathetic reflex [128]. Similar studies that directly record renal sympathetic nerve discharge activity have shown that EA has a therapeutic effect on chronic kidney disease. This effect may be achieved by regulating the activity of the renal sympathetic nervous to lower BP [129].

Through extensive clinical research and animal experiments, it has been found that acupuncture can improve cardiovascular disease through ANS. The pathway of acupuncture signal transduction indicates that the hypothalamus plays a crucial role in regulating ANS. Therefore, we will focus on the hypothalamus and further explore the role of acupuncture in improving cardiovascular disease.

## 5 Current status and analysis of acupuncture modulation of hypothalamus involved in the treatment of cardiovascular diseases

The mechanism underlying the effects of acupuncture at specific acupoints is highly complex [130]. As a form of sensory stimulation, acupuncture activates receptors at acupoints, leading to the generation of nerve impulses that are subsequently transmitted to the CNS [131]. Within the CNS, acupuncture information undergoes integration and processing [132]. To exert therapeutic effects by regulating the action of the neurohumoral system on target organs [133]. Therefore, an in-depth study of the conduction of acupuncture signals in the CNS is a crucial step in revealing its therapeutic mechanism of action. The hypothalamus, despite representing only 0.3 % of the total brain weight, is a pivotal central structure located beneath the cortex that is closely connected to the ANS [134,135]. Clinical studies have shown that heart failure is associated with chronic upregulation of SNS, and acupuncture can inhibit the transduction of nitric oxide signals in the hypothalamus, thereby inhibiting SNS to protect heart function [136].

### 5.1 Effect of acupuncture on neurons in the hypothalamus

The parameter HRV is modulated by the blood-pressure control-system, influences from the hypothalamus, and, in particular, the vagal cardiovascular center in the lower brainstem [137]. The regulatory mechanism of acupuncture on the hypothalamus has been widely confirmed. Clinical studies have found that acupuncture at LR3 (the posterior depression of the first metatarsal space on the lateral side of the dorsum of the foot) acupoint in patients with essential hypertension can significantly reduce BP, by promoting functional connectivity between the hypothalamus, frontal lobe, cerebellum, and insula [138]. Experimental studies have shown that EA at PC6 can inhibit neurons in the dorsomedial hypothalamus and reverse cardiac autonomic function and tachycardia [139]. Another study suggests that EA pretreatment can significantly increase the discharge frequency of lateral hypothalamic area (LHA) excitatory pyramidal neurons and reduce the energy of the local field potential spectrum [140]. Similarly, EA can inhibit glutamatergic neurons in the LHA and alleviate MIRI [141]. Some studies have also found that EA can reduce ST segment elevation, arrhythmia scores, and morphological changes in MIRI cardiomyocytes in rats, as well as decrease the expression of c-fos protein in the nucleus of LHA. In addition, it can also activated GABAergic neurons in LHA and inhibited glutamatergic neurons [142].

The hypothalamus controls several key nuclei of the ANS, such as the key neurons of the SNS that regulate vasomotor tension, which project from the PVN to the RVLM and participate in the activation of the central reflex pathway caused by cardiac mechanoreceptor stimulation [143]. PVN, as an integrated site of the neuroendocrine and ANS systems, participates in the regulation of BP and cardiovascular systems [144,145]. As such, research has focused on how acupuncture can exert a protective effect on cardiac function by modulating sympathetic activity through the hypothalamus. EA activates neurons in the hypothalamic PVN through sensory stimulation and projects them to the RVLM, which is responsible for regulating sympathetic excitability in the cardiovascular region [146]. Cui et al. observed the discharge, SNS discharge, and hemodynamic parameters of PVN neurons and found that the hippocampus- PVN- SNS pathway plays an important role in the treatment of myocardial ischemia by EA, with the key neurons being interneurons [147]. The latest research results indicate that EA pretreatment can inhibit CRH neurons in PVN and alleviate MIRI by inhibiting SNS, protecting heart function [148]. Similarly, EA can activate neurons in the ARC nucleus of the hypothalamus, thereby inhibiting the activity of pre motor SNS in RVLM and inhibiting cardiovascular SNS cells activated by visceral reflex stimulation [149].

Besides, acupuncture can reduce apoptosis of hypothalamic neurons and activate brain regions related to BP regulation to regulate ANS balance, thereby reducing BP [150]. Studies have shown that the central cyclooxygenase and lipoxygenase pathways play a mediating role in the cardiovascular effects induced by orexinergic neurons [151], and orexinergic neurons in the hypothalamus can receive nerve fiber projections from various brain structures. Acupuncture may improve the cardiovascular response induced by hypertension and psychological stress by regulating Orexin neurons [152]. In summary, EA can regulate ANS through multiple nuclei in the hypothalamus, thereby exerting a protective effect on cardiovascular function.

### 5.2 Effects of acupuncture on hypothalamic neurotransmitters and endocrinology

In synaptic transduction, neurotransmitters play the role of "messengers", which are specific chemicals synthesized by nerve cells and released at nerve endings [153]. Experimental research has found that during alcohol withdrawal, EA has an impact on the levels of NE in the hypothalamic pituitary adrenal axis [154]. This indicates that EA regulates norepinephrine in the hypothalamus to protect heart function. The differential control of cardiovascular motor activity by the dorsomedial hypothalamus and the SNS suggests that central 5-hydroxytryptamine (5-HT) receptors efficiently but selectively modulate dorsomedial hypothalamus-evoked cardiovascular responses [155]. EA is related to dopamine secretion in the hypothalamus of myocardial ischemia rats [156]. Research has shown that EA at HT7 acupoint regulates 5-HT levels in the hypothalamus of rats, alleviating acute MIRI [157]. Similarly, EA at PC6 acupoint and HT7 acupoint can alleviate acute myocardial ischemic injury in hyperlipidemic rats, which is related to their promotion of the release of 5-HT in the PVN [158].

EA protects cardiovascular function by inhibiting opioid receptors in PVN, inhibiting sympathetic outflow and sympathetic excitatory cardiovascular responses [159]. Experimental studies have shown that EA works by β- Endorphins regulate cardiovascular activity in PVN [146]. This indicates that PVN opioid mechanism is involved in acupuncture to regulate the response of BP rise. Its mechanism involves the spinal cord pathway stimulated by EA, including the ARC nucleus, which is an important site for opioid neurotransmitter synthesis. ARC is involved in EA, inhibiting visceral nerves and regulating BP [62]. The stimulation of glutamate on its ion receptors is the reason for EA induced ARC activity [160]. After EA activates Arc neurons, they not only release β- Endorphins directly inhibit cardiovascular sympathetic excitatory neurons (Arc- RVLM pathway) in RVLM, and indirectly inhibit RVLM sympathetic excitatory neurons (Arc- NTS- RVLM pathway) by stimulating NTS neurons, thereby producing antihypertensive effects [161].

Orexin is secreted around the fornix of the lateral hypothalamic nucleus [162,163], and orexin has been found to play a role in regulating cardiovascular function [151,164]. Therefore, some scholars have conducted relevant studies and found that acupuncture may activate Orexin in the lateral saphenous nucleus of the hypothalamus, thereby producing analgesic effects and improving cardiovascular disease [165]. In addition, EA may regulate the expression of oxytocin and arginine vasopressin in the hypothalamus, where oxytocin can lower BP, while arginine vasopressin can increase vascular pressure, indicating that acupuncture signals may regulate vascular pressure and protect heart function by secreting neurotransmitters.

In summary, a large number of clinical studies and animal experiments have found that acupuncture can transmit signals to multiple nuclei in the hypothalamus through sensory stimulation, such as PVN and the LHA. By activating or inhibiting related neurons and their receptors and releasing neurotransmitters, acupuncture can reduce the excitability of SNS, enhance the activity of VNS, regulate the balance between SNS and VNS, and adjust HRV to reduce the impact of changes in ANS function on various cardiovascular diseases.

## 6 Discussion

Acupuncture treatment of cardiovascular diseases has always been an important research direction in the field of traditional Chinese medicine. In clinical practice, acupuncture can lower mean arterial pressure and HR by inhibiting the SNS [91]. About 70 % of mild to moderate hypertensive patients who have discontinued antihypertensive drugs have significantly reduced sympathetic nerve outflow through low-frequency, low-intensity EA treatment. After cessation of an eight weeks of once weekly stimulation, systolic blood pressure can decrease for a long period of two to four weeks, and diastolic blood pressure can also decrease to a small extent, lasting up to a month [113]. Current data have shown that acupuncture play a role in the treatment of hypertension [166]. Although there are differences in the research methods and quality included, this paper provides the data of changes in blood pressure and heart rate in response to acupuncture in physiological and pathological conditions, summarized in Table 1, Table 2, Table 3, Table 4, and conducted a meta-analysis of these data (Figs. S1–6).

**Table 1:** Comparison of SBP and DBP between acupuncture group and other control groups.

Reference	Model	Acupoints	Intervention parameters	Duration	mean difference (95%CI) SBP mmHg	mean difference (95%CI) DBP mmHg
acupuncture group vs sham acupuncture group						
[167]Ma, J et al.	hypertension	auricular acupuncture	MA		−7.88 (−2.94, −12.81)	−5.85 (−3.85, −9.63)
[168]Yang et al.	hypertension		MA	10 weeks	−3.4(-6.0, −0.9)	−2.0(-3.6, 0.3)
[169]Zheng et al.	mild hypertension		2HZ, 2 mA	9 weeks	−3.3(-0.2, −6.3)	
[170]Kim et al.	hypertension				−4.23(-6.47, −1.99)	−2.53(-3.99, −1.08)
acupuncture group vs untreated group						
[171]Liu et al.	hypertension	ST36, PC6, LR3, SP4, LI11	MA	4 weeks	−8.6(-16.3, −0.8)	−7.8(-12.8, −2.8)
acupuncture group vs. Chinese herbal medicine						
[170]Kim et al.	hypertension				−6.46(-8.04, −4.87)	−3.07(-4.17, −1.96)
[172]Lee et al.	hypertension	LR3, LI11, GB20	MA	6 weeks	−5.00(-12.00, 1.00)	−3.00(-6.00, 0.00)


CI: confidence interval; DBP: diastolic blood pressure; SBP: systolic blood pressure.

**Table 2:** Physiological and pathological changes in SBP before and after acupuncture.

Reference	Model	Acupoints	Intervention parameters	Duration	pre-acupuncture SBP mmHg	post-acupuncture SBP mmHg
[138]Zheng et al.	hypertension	LR3	MA	2 weeks	134.93 ± 9.05	129.71 ± 8.66
[173]Zheng et al.	hypertension		3HZ, 2 mA	8 weeks	171.93 ± 21.66	140.03 ± 10.86
[174]Hao et al.	hypertension	KI1, LR3	moderate stimulation	7 days	155.27 ± 10.11	129.40 ± 13.31
[175]Zhao et al.	hypertension	CV4, ST36, ST40, SP6, LR3	MA	40 days	164.26 ± 32.25	129.01 ± 36.00
[176]Ma et al.	hypertension	LI11	20HZ	15 days	153.70 ± 13.01	139.22 ± 9.64
[177]Yang et al.	hypertension	LI11, LR3	2HZ/100HZ 10–20 mA	2 weeks	136.03 ± 9.96	121.36 ± 9.10
[178]Abdi et al.	hypertension	ST25, RN4 SP6	30-40HZ	6 weeks	122.9 ± 25.80	112.9 ± 20.20
[179]Palma et al.	climacteric symptoms		MA	3 months	122.7 ± 9.00	115.3 ± 6.30
[180]Migliarese et al.	Arterial Hypertension	CV4, LV3, LI11, ST36	MA	6 weeks	131.1 ± 10.70	126.0 ± 10.10
[181]Zheng	hypertension	LI11, ST36, ST40, SP6	MA	4 weeks	151.29 ± 4.70	140.08 ± 6.19
[182]Kenichi et al.	mild hypertension	PC6, LI4 ST36, LR3, GV20	MA	once	133.9 ± 0.3	128.9 ± 1.8
[182]Kenichi et al.	heath	PC6, LI4, ST36, LR3, GV20	MA	once	111.4 ± 0.4	111.2 ± 1.7
[183]Zhao et al.	stroke	PC6, GV26, SP6	MA	6 weeks	154.72 ± 14.64	131.90 ± 11.75
[184]Zhang et al.	hypertension	LR3, KI3	MA	2 weels	136.3 ± 17.43	130.0 ± 13.44
[185]Kim et al.	hypertension	ST36, PC 6	MA	8 weeks	139.88 ± 10.72	142.88 ± 8.13
[186]Sokunbi et al.	heath	PC5, PC6, ST36, LI4	MA	once	121 ± 10.69	111.5 ± 9.24
[187]Tao et al.	cerebral hemorrhage	LI11, PC6, ST36, LR3	MA	Fourth 24h	151.1 ± 20.8	147.3 ± 21.6
[188]Li et al.	essential hypertension	ST9, LI11, LI4, ST36, LR3	MA	6 weeks	145.59 ± 11.90	133.76 ± 9.61
[188]Li et al.	essential hypertension	ST9, LI4, LR3, ST36, LI11	MA	8 weeks	155.9 ± 8.9	129.5 ± 9.4
[189] Terenteva et al.	hypertension	ST36, ST37, PC5, PC6, LR3, SP4	MA	8 weeks	150 ± 2	140 ± 3
[190]Huang et al.	Posterior circulation ischemia	Stellate Ganglion	MA	2 weeks	150.06 ± 8.43	131.86 ± 5.93
[190]Huang et al.	Posterior circulation ischemia	Stellate Ganglion	MA	2 weeks	148.92 ± 7.36	138.97 ± 8.49
[191]Chen et al.	hypertension	LI11, ST40	MA	2 weeks	166.03 ± 17.07	135.60 ± 12.42
[192]Gao et al.	hypertension	ST9, LI4, LR3, ST36, LI11	MA	6 weeks	160.76 ± 7.71	130.41 ± 10.15
[193]Huang et al.	heath	ST36	Lifting-thrusting	once	117.87 ± 8.37	106.67 ± 7.75
[193]Huang et al.	heath	ST36	Twisting-rotating	once	112.67 ± 10.26	106.53 ± 9.52

SBP: systolic blood pressure.

**Table 3:** Physiological and pathological changes in DBP before and after acupuncture.

Reference	Model	Acupoints	Intervention parameters	Duration	pre-treatment DBP mmHg	post-treatment DBP mmHg
[138]Zheng et al.	hypertension	LR3	MA	2 weeks	86.29 ± 7.35	86.71 ± 6.11
[173]Zheng et al.	hypertension		3HZ, 2 mA	8 weeks	97.20 ± 12.70	87.47 ± 6.47
[174]Hao et al.	hypertension	KI1, LR3	moderate stimulation	7 days	89.07 ± 8.38	75.07 ± 9.95
[175]Zhao et al.	hypertension	CV4, ST36, ST40, SP6, LR 3	MA	40 days	96.01 ± 13.50	81.01 ± 12.00
[176]Ma et al.	hypertension	LI11	20HZ	15 days	96.82 ± 6.32	89.70 ± 5.46
[177]Yang et al.	hypertension	LI11, LR3	2HZ-100HZ 10–20 mA	2 weeks	91.50 ± 9.76	75.18 ± 8.90
[178]Abdi et al.	hypertension	ST25, RN4, SP6	30-40HZ	6 weeks	68.1 ± 11.20	64.70 ± 10.7
[179]Palma et al.	climacteric symptoms		MA	3 months	77.3 ± 4.00	68.90 ± 5.00
[180]Migliarese et al.	Arterial Hypertension	CV4, LV3, LI11, ST36	MA	6 weeks	85.30 ± 9.10	82.10 ± 7.50
[181]Zheng	hypertension	LI11, ST36, CV4, SP6	MA	4 weeks	92.03 ± 2.95	83.89 ± 4.01
[182]Kenichi et al.	mild hypertension	PC6, LI4, ST36, LR3, GV20	MA	once	85.8 ± 0.3	82.8 ± 1.5
[182]Kenichi et al.	normotensive patients	PC6, LI4, ST36, LR3, GV20	MA	once	70.8 ± 0.3	70.7 ± 1.4
[183]Zhao et al.	stroke	PC6, GV26, SP6	MA	6 weeks	88.23 ± 7.76	78.62 ± 5.49
[184]Zhang et al.	hypertension	LR3, KI3	MA	2 weels	89.50 ± 8.43	87.9 ± 5.04
[185]Kim et al.	hypertension	ST36, PC 6	MA	8 weeks	94.11 ± 7.80	95.16 ± 6.20
[186]Sokunbi et al.	heath	PC5, PC6, ST36, LI4	MA	once	76.0 ± 8.96	70.5 ± 0.24
[187]Tao et al.	cerebral hemorrhage	LI11, PC6, ST36, LR3	MA	one day	85.5 ± 10.5	87.5 ± 12.4
[188]Li et al.	essential hypertension	ST9, LI11, LI4, ST36, LR3	MA	6 weeks	85.25 ± 6.85	80.27 ± 6.43
[188]Li et al.	essential hypertension	ST9, LI4, LR3, ST36, LI11	MA	8 weeks	78.3 ± 7.1	72.8 ± 5.6
[189]Terenteva et al.	hypertension	ST36, ST37, PC5, PC6, LR3, SP4	MA	8weeks	85 ± 2	79 ± 3
[190]Huang et al.	Posterior circulation ischemia	Stellate Ganglion	MA	2 weeks	90.13 ± 5.71	75.38 ± 4.93
[190]Huang et al.	Posterior circulation ischemia	Stellate Ganglion	MA	2 weeks	88.62 ± 6.38	80.26 ± 6.21
[191]Chen et al.	hypertension	LI11, ST40	MA	2 weeks	86.94 ± 12.42	77.74 ± 8.46
[192]Gao et al.	hypertension	ST9, LI4, LR3, ST36, LI11	MA	6 weeks	95.26 ± 4.16	81.76 ± 8.26
[193]Huang et al.	heath	ST36	Lifting-thrusting	once	69.33 ± 8.93	65.87 ± 8.61
[193]Huang et al.	heath	ST36	Twisting-rotating	once	64.8 ± 8.16	65.4 ± 8.55
[194]Okada et al.	healthy men	LI10	MA	once	62.2 ± 2.0	58.6 ± 2.4
[195] Nakaharaet al.	healthy volunteers	PC4	1V, 1HZ	once	86.6 ± 2.9	81.4 ± 2.3

DBP: diastolic blood pressure.

**Table 4:** Physiological and pathological changes in HR before and after acupuncture.

Reference	Model	Acupoints	Intervention parameters	Duration	pre- acupuncture HR (bpm)	post- acupuncture HR (bpm)
[85]Li et al.	under fatigue	LI4, PC6	MA	once	77.42 ± 6.15	64.60 ± 6.10
[85]Li et al.	heath	LI4, PC6	MA	once	78.00 ± 7.75	65.41 ± 6.80
[98]Takahashi et al.	health	near the left cervical ganglia	2HZ no pain	2 weeks	64.73 ± 8.08	62.67 ± 8.63
[100]Kurono et al.	healthy men	CV17	MA	two days	73.5 ± 9.3	70.8 ± 9.1
[174]Hao et al.	elevated BP	KI1, LR3	MA	7 days	78.13 ± 10.26	69.23 ± 5.66
[182]Kimura et al.	heath	PC6, LI4, ST36, LR3, GV20	MA	once	63.3 ± 0.2	62.9 ± 0.3
[182]Kimura et al.	mild hypertension	PC6, LI4, ST36, LR3, GV20	MA	once	68.9 ± 0.2	68.4 ± 0.3
[186]Sokunbi et al.	heath	PC5, PC6, ST36, LI4	MA	once	67.70 ± 5.29	66.9 ± 4.80
[189]Terenteva et al.	hypertension	ST36, ST37, PC5, PC6, LR3, SP4	MA	8 weeks	68 ± 2	67 ± 2
[193]Huang et al.	heath	ST36	Lifting-thrusting	once	73.9 ± 9.9	68.9 ± 6.0
[193]Huang et al.	heath	ST36	Twisting-rotating	once	78.9 ± 13.3	74.3 ± 10.3
[194]Okada et al.	healthy men	LI10	MA	once	60.2 ± 1.4	55.9 ± 1.6
[195]Nakahara et al.	healthy volunteers	PC4	1V, 1HZ	once	66.2 ± 2.0	62.7 ± 1.7
[196]Kenji et al.	heath	LI10	supine	once	64 ± 8.6	60.4 ± 8.7
[196]Kenji et al.	heath	LI10	sitting	once	73.6 ± 9.6	66.7 ± 8.7
[197]Satoh et al.	healthy male	GV20	MA	Once	67.0 ± 2.6	59.8 ± 2.4
[198]Takayama et al.	healthy volunteers	LR3	MA	once	67.3 ± 10.1	64.2 ± 8.8
[199]Diao et al.	Myocardial ischemia	PC6	EA	4 weeks	82.0 ± 9.0	73.0 ± 7.0

HR: heart rate.

At present, research on acupuncture regulation of ANS has gained consensus among the world [90], but the pivot mechanism is not yet very clear. As the two fundamental organs of human life, the functions of the brain and heart are closely related [200]. Many clinical studies have confirmed the potential mechanisms by which the brain is involved in regulating cardiovascular disease [201]. EA, as a sensory stimulus, acts on the surface of the body and is transmitted to the CNS [202]. The hypothalamus [203], as the most advanced center for controlling visceral activity, including many important neural nuclei, which are associated with tissues such as the hippocampus and brainstem, and have a high degree of regulation and integration on the ANS [204]. In recent years, the study of hypothalamic functional networks through acupoint stimulation has received widespread attention [138]. Functional magnetic resonance imaging technology can more intuitively observe the brain effects after acupuncture. Using this technology, it was found that acupuncture at HT7 acupoint can specifically activate the limbic systems such as the frontal lobe, cerebellum, hippocampus, thalamus, and insula in the brain [205]. Acupuncture stimulation can activate the limbic system and somatosensory brain regions, and mediate their effects by mobilizing the relevant functional networks of the brain.

During the low-frequency and low-intensity stimulation of MA or EA, the nerves regulating cardiovascular function are usually located above the nerves in the upper and lower limbs [195,206,207]. Bäcker M et al. found that human hand, arm, and leg acupoints do indeed trigger cardiovascular responses through acupuncture and/or EA stimulation [208]. The stimulation of these acupoints can activate potential sensory nerve pathways, transmit signals to multiple regions in the CNS, and ultimately regulate cardiovascular function by regulating autonomic nervous outflow. The excitability and inhibitory neurotransmitters of CNS may form the basis for regulating somatic cell input and autonomic nerve outflow during EA.

Acupuncture has a slow effect on ANS, but its duration is relatively prolonged, usually exceeding the stimulation period [166,182,184,209]. It is commonly used in clinical acupuncture pretreatment to prevent cardiovascular diseases [210]. The therapeutic effect of acupoints is reflected in the transduction of signals to the brain, through different acupuncture techniques, or appropriate EA intensity and frequency, after local acupuncture [211]. The brain, as a high-level center, integrates acupuncture signals and transmits them to the hypothalamus, thereby regulating ANS and improving visceral function [212]. Therefore, this review comprehensively elaborates on the process of acupuncture sensory signal transduction to the hypothalamus, summarizes the research progress of ANS and hypothalamus in acupuncture treatment of cardiovascular diseases, and explores the signal transduction pathways and related neurophysiological mechanisms of acupuncture regulation of cardiovascular diseases.

With the deepening of scientific research, more and more new experimental methods are applied in the field of acupuncture. Researchers have applied optogenetic techniques to regulate NTS and confirmed its involvement in primary motor cortex EA for post-stroke dysphagia [213]. Some scholars have also found through multichannel in vivo neuroelectric recordings that EA pretreatment can alleviate MIRI by regulating the PVN-IN neural pathway [214]. This also provides effective assistance for acupuncture research to reveal the central mechanism. A study has recorded the effects of EA on GABAergic neurons in transgenic mice using vitro patch clamp technology [215]. Similarly, single-cell sequencing technology has also been used in the study of acupuncture mechanisms. Scholars have used this technique to investigate the effects of acupuncture on the expression of CA1 and miRNA in the hippocampus and entorhinal cortex of MCAO rats [216]. In recent research, metabonomic analysis technology has also been applied that acupuncture effectively modulated bile acids metabolism in spontaneously hypertensive rats renal cortex tissues to exert a hypotensive effect [217]. The application of patch clamp technology, single-cell sequencing and metabolomics indicates that acupuncture research has taken an important step towards in vitro and cellular levels.

The hypothalamus and ANS are important targets of acupuncture treatment. They play a key role in mediating the role of acupuncture and restoring the balance of the body. It is necessary to conduct systematic research in order to determine the patterns and mechanisms of specific CNS and corresponding organs that affect the ANS. This has important theoretical and practical significance for in-depth research on the central peripheral integration mechanism of acupuncture in treating cardiovascular diseases.

The effect characteristics of acupuncture are specific and closely related to the acupoints, methods, and parameters stimulated. Recently, research using optogenetic techniques has revealed that EA can modulate the neuroanatomical mechanisms of the vagus-adrenal axis, indicating that different acupuncture depths, frequencies, and current intensities may exert distinct effects on the ANS [41]. However, there is currently limited research on the intensity and frequency of EA stimulation to improve cardiac function, lacking a unified standard, and further research is needed to explore. In addition, a study suggests that MA can regulate BP in spontaneously hypertensive rats, change the glucose metabolism of the PVN, and affect the mRNA and protein expression levels of differentially expressed genes in the PVN [218]. This indicates that MA reduces BP by regulating various biological processes and genes/proteins of PVN.

In recent years, acupuncture studies combined with optogenetic techniques to modulate target neurons and the application of multichannel in vivo neuroelectric recordings have revealed a functional link between acupuncture signals and neurons in multiple brain regions in cardiovascular disease [215,219]. Similarly, in vitro patch clamp technology is a neuroelectrical recording method that analyzes the regulatory mechanism of acupuncture on the CNS to the periphery from another perspective [220,221]. On the other hand, under physiological and pathological conditions, acupuncture treatment of cardiovascular diseases usually has multi-level characteristics. Although acupuncture has been studied in the field of neural pathways, the exploration of molecular mechanisms in the peripheral and CNS is still not in-depth enough. With the maturity of emerging technologies such as single-cell sequencing [222,223] and metabolomics [[224], [225], [226]], more advanced technological means have been provided to explore the molecular mechanisms of nerves. At present, the connection between glial cells in the central and peripheral nervous systems has become the focus of current research. However, it is still unclear how glial cells participate in the central anti-inflammatory effect of acupuncture in cardiovascular diseases. In addition, we need to further explore the paracrine effect of glial cells mediated by acupuncture on neurons, as well as the mechanism of synaptic modification and reconstruction on neurons to protect cardiac function. Therefore, in order to deeply explore the mechanism of the effects between the meridians and organs, as well as the central and peripheral nervous systems, we need more rigorous and objective basis to promote the development of acupuncture and better serve clinical practice.

## Funding statement

Rong-lin Cai was supported by the 10.13039/501100001809National Natural Science Foundation of China 82074536, 10.13039/501100003995Natural Science Foundation of Anhui Province 2108085Y30, Distinguished Young Youth Scientific Research Project in Universities of Anhui Province 2022AH020043, Research Funds of Center for Xin'an Medicine and Modernization of Traditional Chinese Medicine of IHM 2023CXMMTCM019.

Qing Yu was supported by 10.13039/501100001809National Natural Science Foundation of China 82104999, 10.13039/501100003995Natural Science Foundation of Anhui Province 2108085QH364, Excellent Young Youth Scientific Research Project in Universities of Anhui Province 2022AH030062, Opening project of key laboratory related to meridians and organs AHMVC2024001.

## Data availability statement

There is no relevant research data deposited into a publicly available repository for this review. No data was used for the research described in the article.

## CRediT authorship contribution statement

**Xiang Zhou:** Writing – original draft. **Jie Zhou:** Writing – original draft. **Fan Zhang:** Writing – review & editing. **Qi Shu:** Writing – review & editing. **Yan Wu:** Writing – review & editing. **Hui-min Chang:** Writing – review & editing. **Bin Zhang:** Writing – review & editing. **Rong-lin Cai:** Writing – review & editing. **Qing Yu:** Writing – review & editing.

## Declaration of competing interest

The authors declare that they have no known competing financial interests or personal relationships that could have appeared to influence the work reported in this paper.
