# Supplementary material for: Key targets of signal transduction neural mechanisms in acupuncture treatment of cardiovascular diseases: Hypothalamus and autonomic nervous system
Source: Heliyon. 2024 Sep 20;10(19):e38197. doi: 10.1016/j.heliyon.2024.e38197 (PMC11462008; doi:10.1016/j.heliyon.2024.e38197)
Supplement: Multimedia component 1 [file mmc1.docx]

**Supplementary information**

**Key targets of signal transduction neural mechanisms in acupuncture treatment of cardiovascular diseases: hypothalamus and autonomic nervous system**

Xiang Zhou^a,e,1^, Jie Zhou^a,1^, Fan Zhang^a^, Qi Shu^a^, Yan Wu^a^, Hui-min Chang^a^, Bin Zhang^a^, Rong-lin Cai^b,c,d,^^[[1]](#footnote-1)^*, and Qing Yu^a,b,c,^^[[2]](#footnote-2)^**

*^a^College of Acupuncture and moxibustion, Anhui University of Chinese Medicine, Hefei 230038, Anhui Province, China;*

*^b^Institute of Acupuncture and Meridian Research, Anhui Academy of Chinese Medicine, Hefei 230038, Anhui Province, China;*

*^c^Anhui Province Key Laboratory of Meridian Viscera Correlationship, Hefei 230038, China;*

*^d^Center for Xin'an Medicine and Modernization of Traditional Chinese Medicine of IHM, Hefei 230038, China;*

*^e^Anhui Wannan Rehabilitation Hospital (The Fifth People's Hospital of Wuhu), Wuhu 241000, Anhui Province, China*

**Supplementary data**


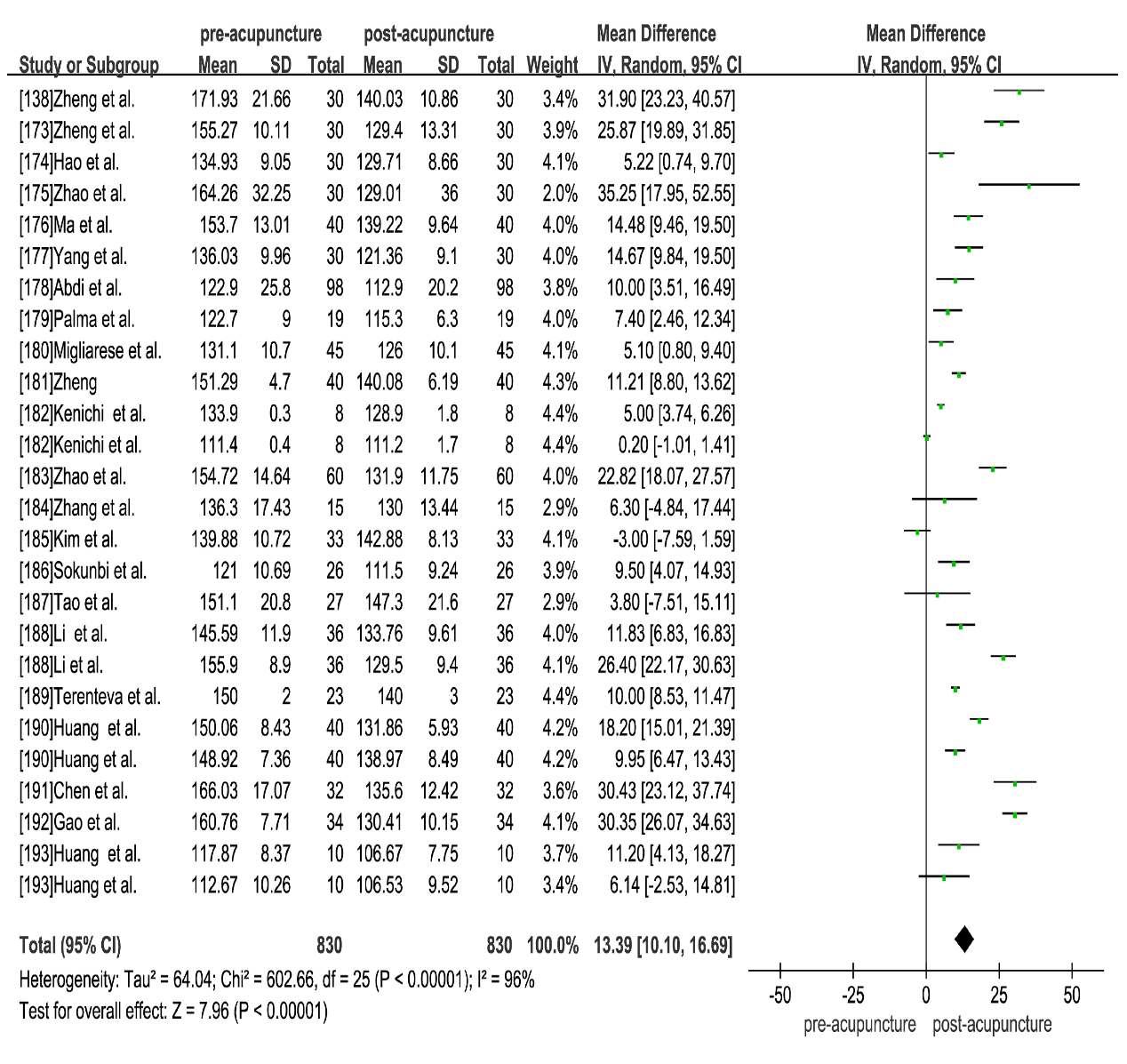


Figure S1. The forest plot of outcome measure ‘the SBP of pre-acupuncture and post-acupuncture.’


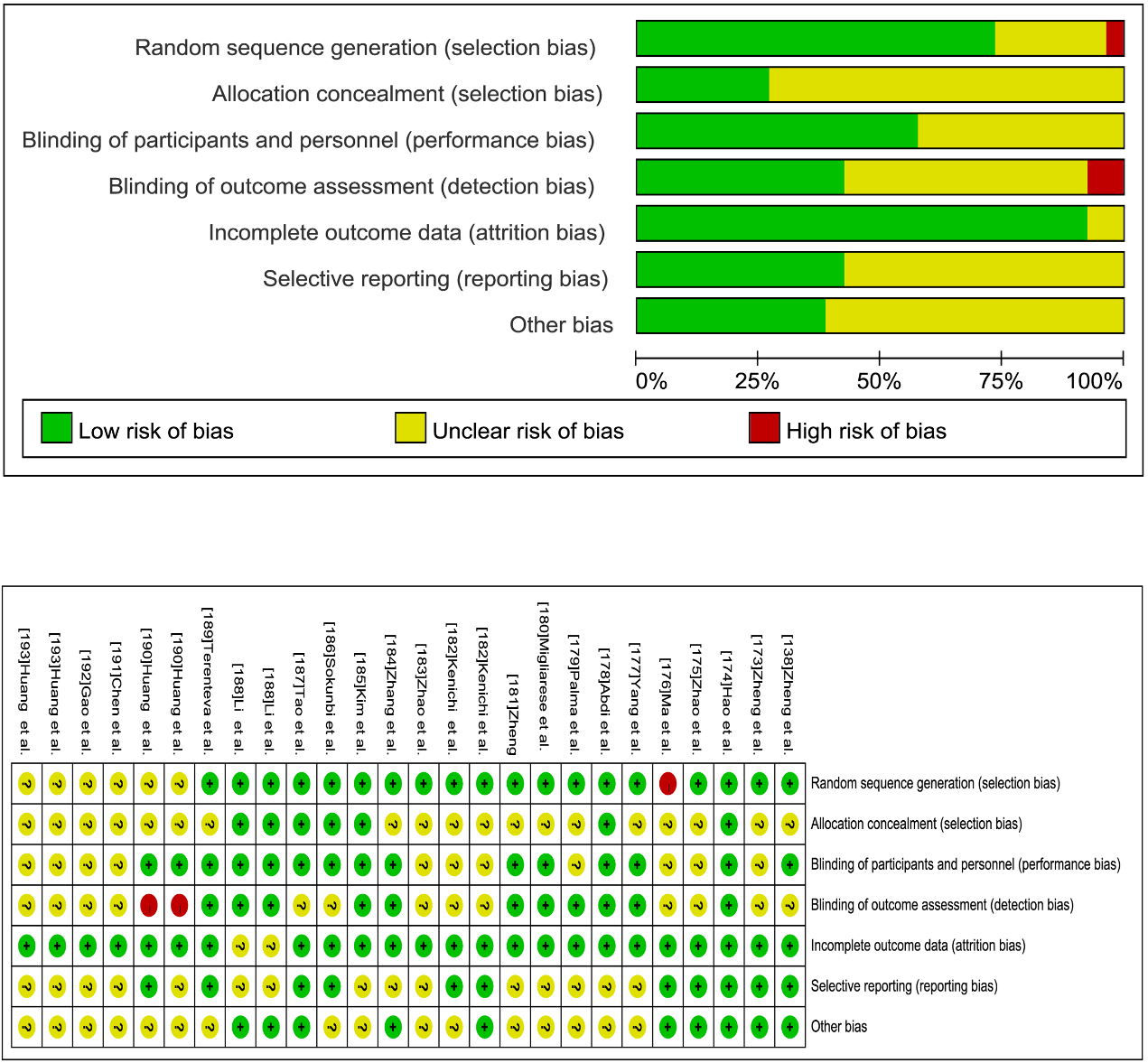


Figure S2. Risk of bias summary and the risk of bias assessment of SBP of pre-acupuncture and post-acupuncture.


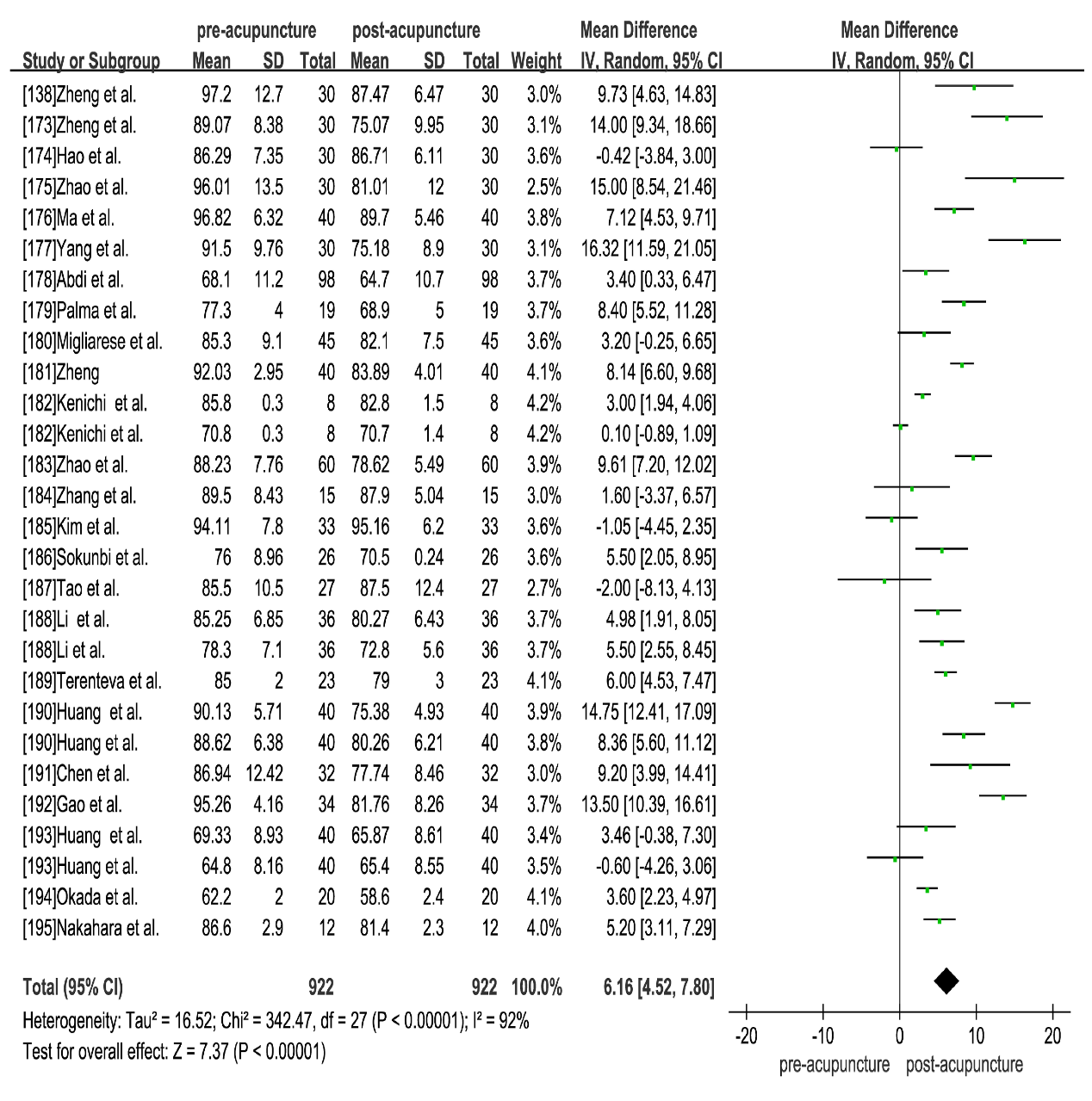


Figure S3. The forest plot of outcome measure ‘the DBP of pre-acupuncture and post-acupuncture.’


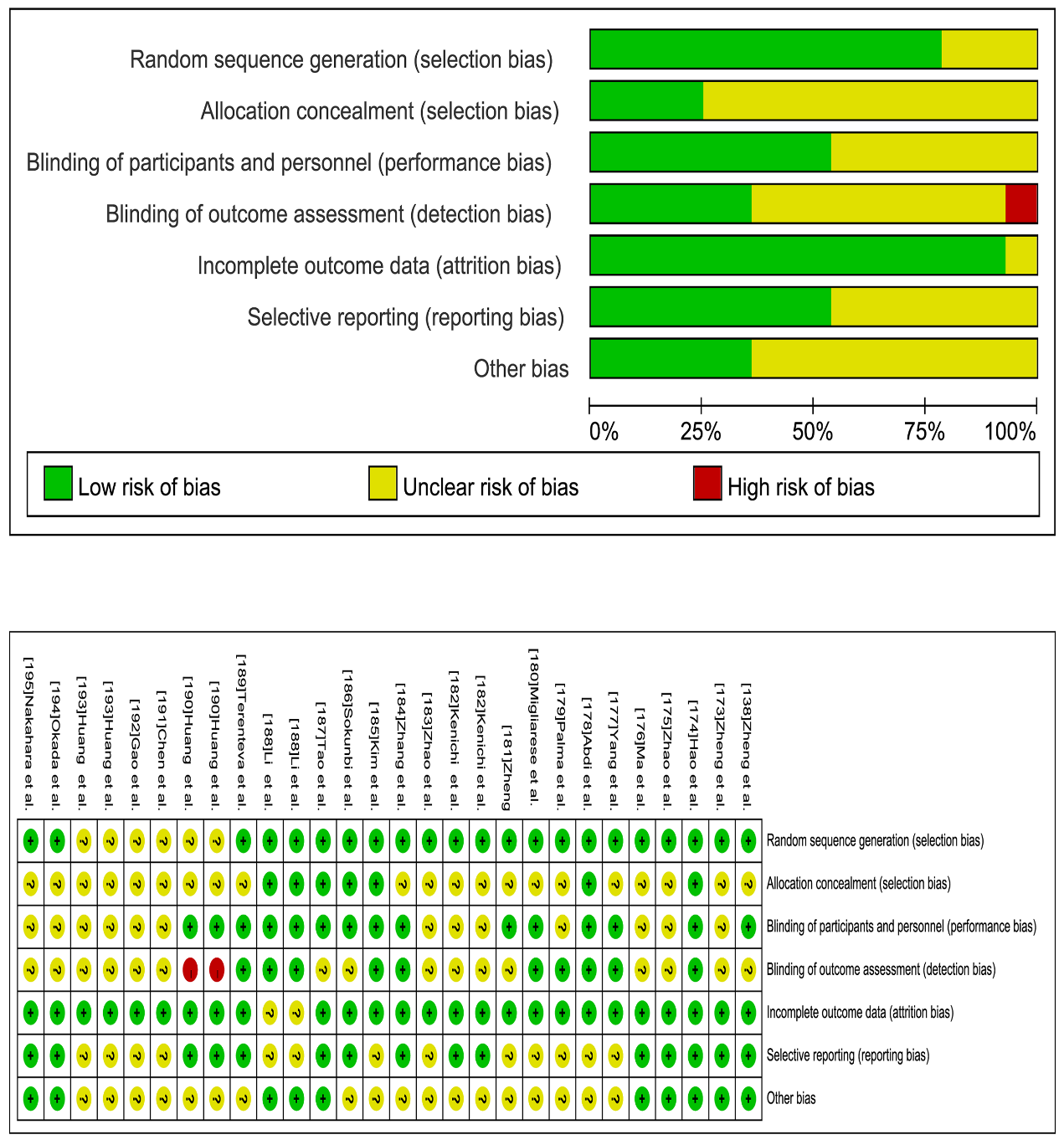


Figure S4. Risk of bias summary and the risk of bias assessment of DBP of pre-acupuncture and post-acupuncture.


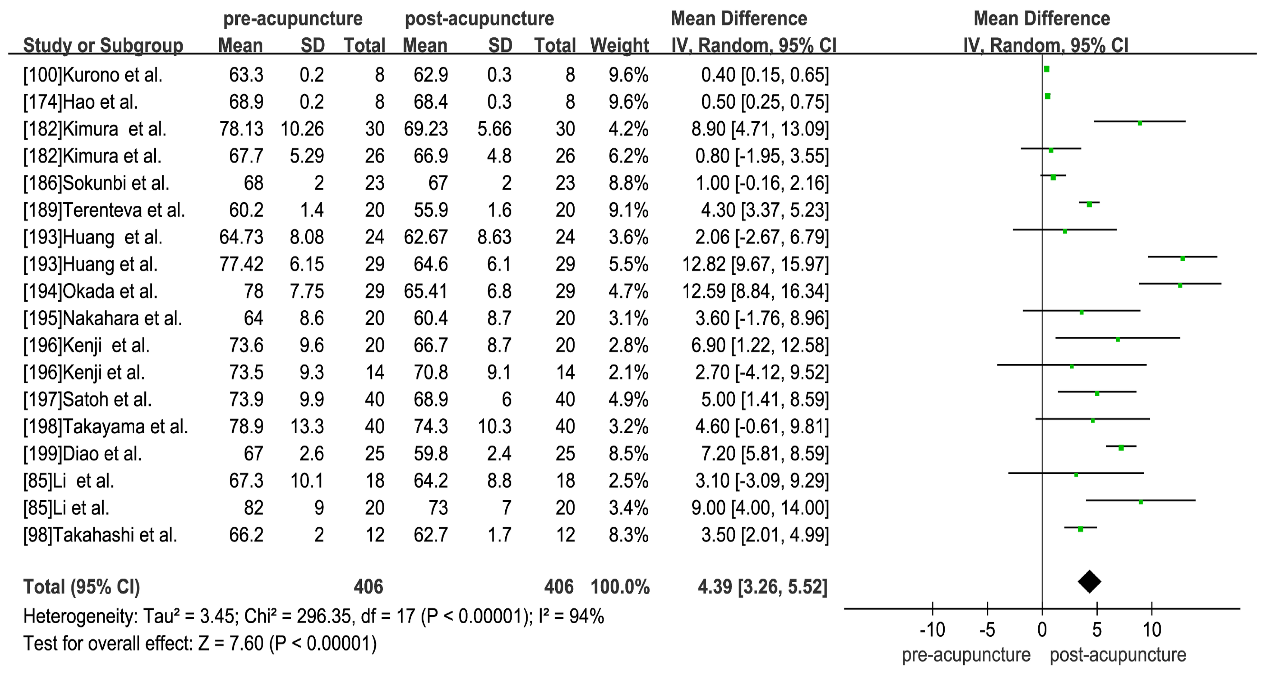


Figure S5. The forest plot of outcome measure ‘the HR of pre-acupuncture and post-acupuncture.’


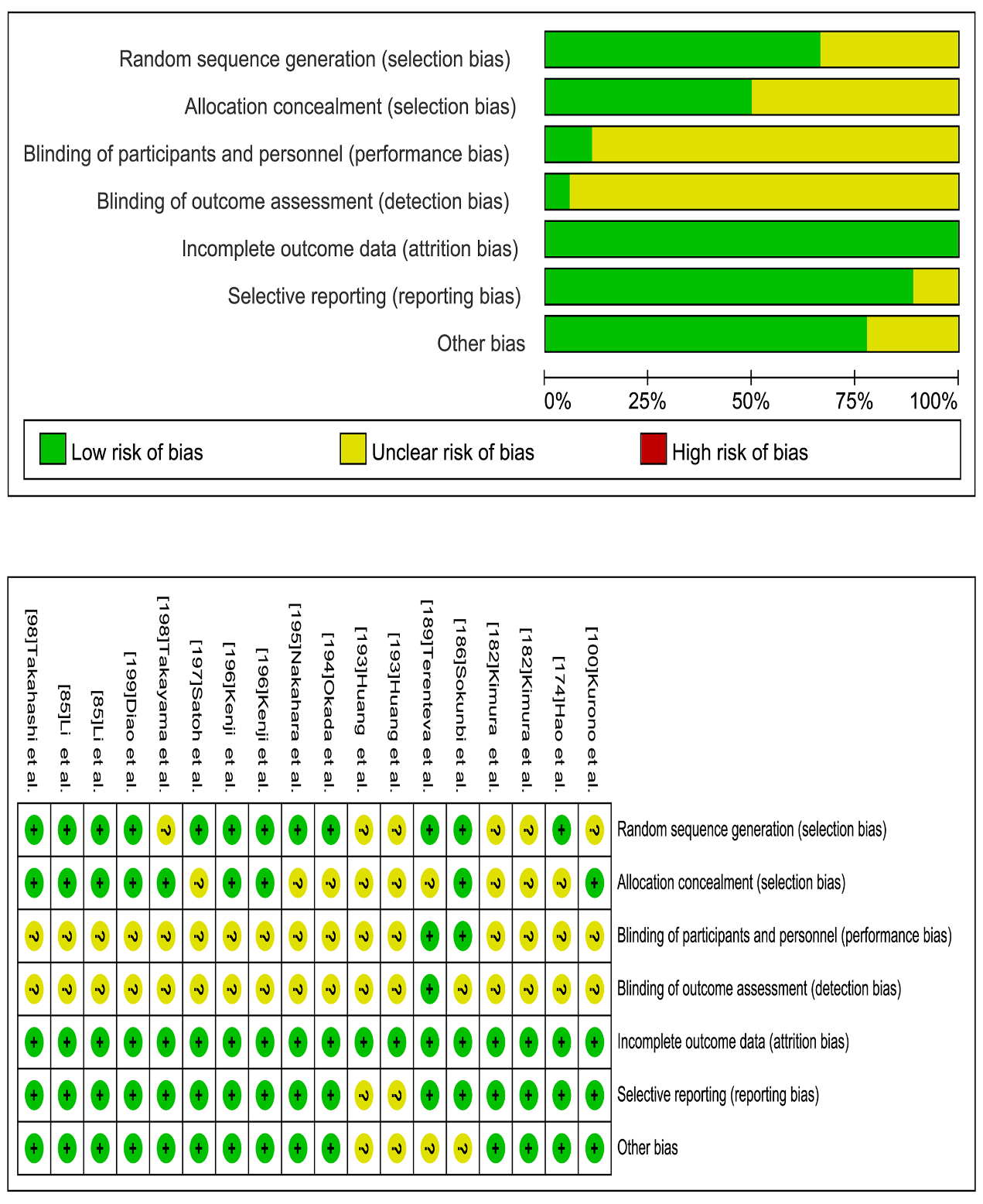


Figure S6. Risk of bias summary and the risk of bias assessment of HR of pre-acupuncture and post-acupuncture.

1. E-mail: [yuqing@ahtcm.edu.cn](mailto:yuqing@ahtcm.edu.cn) (Q. Yu), [ronglincai@ahtcm.edu.cn](mailto::ronglincai@ahtcm.edu.cn) (R.-l. Cai).

   ^1^Xiang Zhou and ^1^Jie Zhou have contributed equally to this article. [↑](#footnote-ref-1)
2. * Corresponding author. Institute of Acupuncture and Meridian Research, Anhui Academy of Chinese Medicine, No. 103 Meishan road, Shushan district, Hefei, 230038, Anhui province, China.

   ** Corresponding author. Institute of Acupuncture and Meridian Research, Anhui Academy of Chinese Medicine, No. 103 Meishan road, Shushan district, Hefei, 230038, Anhui province, China [↑](#footnote-ref-2)
